# Utilizing Benzotriazole and Indacenodithiophene Units to Construct Both Polymeric Donor and Small Molecular Acceptors to Realize Organic Solar Cells With High Open-Circuit Voltages Beyond 1.2 V

**DOI:** 10.3389/fchem.2018.00147

**Published:** 2018-05-01

**Authors:** Ailing Tang, Fan Chen, Bo Xiao, Jing Yang, Jianfeng Li, Xiaochen Wang, Erjun Zhou

**Affiliations:** ^1^CAS Key Laboratory of Nanosystem and Hierarchical Fabrication, CAS Center for Excellence in Nanoscience, National Center for Nanoscience and Technology, Beijing, China; ^2^University of Chinese Academy of Sciences, Beijing, China

**Keywords:** benzotriazole, indacenodithiophene, fullerene-free organic solar cells, high open-circuit voltage, non-fullerene acceptor

## Abstract

Devolopment of organic solar cells with high open-circuit voltage (*V*_OC_) and power conversion efficiency (PCE) simutaniously plays a significant role, but there is no guideline how to choose the suitable photovoltaic material combinations. In our previous work, we developed “the Same-Acceptor-Strategy” (SAS), by utilizing the same electron-accepting segment to construct both polymeric donor and small molecular acceptor. In this study, we further expend SAS to use both the same electron-accepting and electron-donating units to design the material combination. The p-type polymer of PIDT-DTffBTA is designed by inserting conjugated bridge between indacenodithiophene (IDT) and fluorinated benzotriazole (BTA), while the n-type small molecules of BTA*x* (*x* = 1, 2, 3) are obtained by introducing different end-capped groups to BTA-IDT-BTA backbone. PIDT-DTffBTA: BTA*x* (*x* = 1–3) based photovolatic devices can realize high *V*_OC_ of 1.21–1.37 V with the very small voltage loss (0.55–0.60 V), while only the PIDT-DTffBTA: BTA3 based device possesses the enough driving force for efficient hole and electron transfer and yields the optimal PCE of 5.67%, which is among the highest value for organic solar cells (OSCs) with a *V*_OC_ beyond 1.20 V reported so far. Our results provide a simple and effective method to obtain fullerene-free OSCs with a high *V*_OC_ and PCE.

**Graphical abstract:**
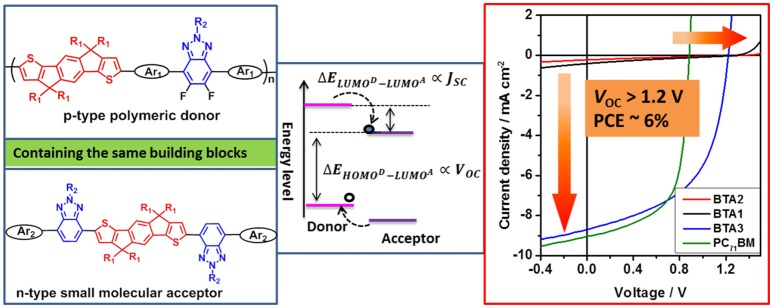
We applied a simple design concept to realize organic solar cells with ultrahigh open-circuit voltage beyond 1.2 V and power conversion efficiency of 5.67%, by utilizing the same building blocks of indacenodithiophene and benzotriazole to design both p-type polymeric donor and n-type small molecular acceptors.

## Introduction

As one of the most promising technique in photoelectric conversion, bulk-heterojunction (BHJ) organic solar cells (OSCs) have been extensively studied. For a long time, fullerene derivatives have taken up the major part of the acceptor materials, benefited from their high electron affinity and electron mobility, as well as isotropic charge transport. (Guldi, 2000; von Hauff et al., 2005; Anthony et al., 2010; Eftaiha et al., 2014) It's not until the recent 2 years that non-fullerene small molecular acceptors (NFSMAs) with strong sunlight harvesting capability and tunable energy levels have drawn considerable attention and hundreds of novel NFSMAs have been developed (Hwang et al., 2015; Lin et al., 2015, 2016a,b; Zhong et al., 2015; Guo et al., 2016; Holliday et al., 2016; Li et al., 2016b, 2017; Wu et al., 2016; Duan et al., 2017a,b; Fan et al., 2017; Liu et al., 2017; Sun et al., 2017; Wang et al., 2017; Xiao B. et al., 2017a,b; Xu S. J. et al., 2017; Xu X. et al., 2017; Yang et al., 2017; Zhang G. et al., 2017; Zhang Z. G et al., 2017) To date, the power conversion efficiencies (PCEs) of the fullerene-free OSCs have reached up to 13%. (Xiao Z. et al., 2017a; Zhao et al., 2017). The short circuit current (*J*_SC_) and fill factor (FF) in these efficient fullerene-free OSCs could arrive as high as 18–25 mA cm^−2^ and 60–70%, respectively. However, the open-circuit voltage (*V*_OC_) values remain relatively low (<1.0 V), because of the large energy loss (*E*_loss_) (Xiao Z. et al., 2017b).

**Figure 1:**
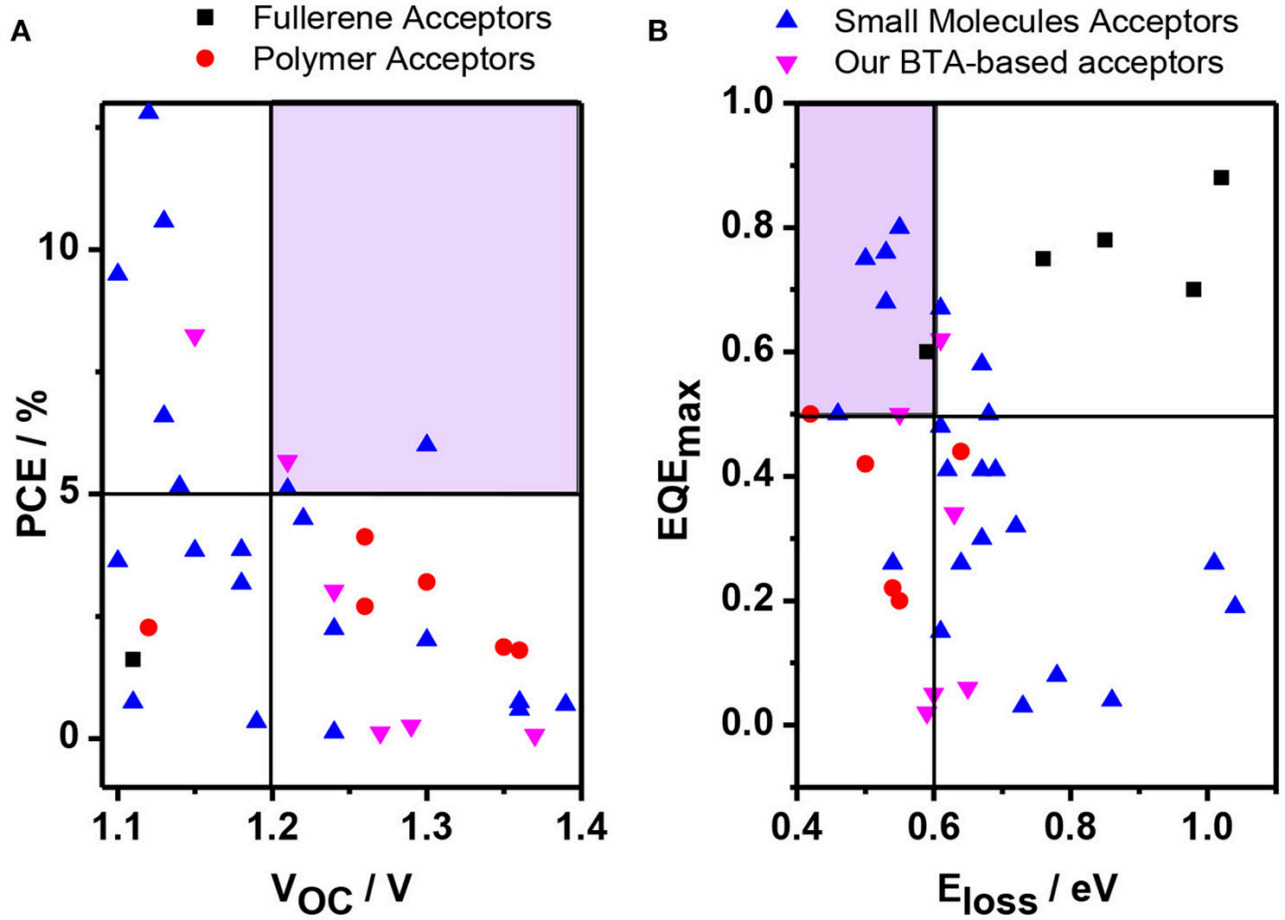
**(A)** Plots of PCE against *V*_OC_ as well as **(B)** EQE_max_ against E_loss_ in various OSCs with fullerene and non-fullerene acceptors with the *V*_OC_ > 1.1 V. The according references were shown in Supporting Information.

Recently, by developing novel NFSMAs with high LUMO levels and choosing suitable p-type polymers, the resulted solar cells could realize high *V*_OC_ values of beyond 1.0 V (Yang et al., 2014; Yu et al., 2014; Zhang et al., 2015, 2016; Baran et al., 2016; Fu et al., 2016; Li et al., 2016a; Liu et al., 2016; Ni et al., 2016; Chen et al., 2017; Ding et al., 2017; Xiao B. et al., 2017a; Zhan et al., 2017; Zhang Y. et al., 2017). In fact, there is always a problematic trade-off between *J*_SC_ and *V*_OC_. Thus, very limited devices could simultaneously realize a high *V*_OC_ of beyond 1.2 V and a high PCE (As shown in Figure 1) (Fu et al., 2016; Xiao B. et al., 2017a; Zhan et al., 2017; Zhang Y. et al., 2017). For example, Xie et al. reported that the OSCs containing poly(3-hexylthiophene) (P3HT) as electron donor and the oligomer F4TBT4 with four repeated fluorene and di-2-thienyl benzothiadiazole units as electron acceptor can output a high *V*_OC_ above 1.2 V and a PCE of 4.12% (Fu et al., 2016). Our group synthesized a novel benzotriazole based acceptor, BTA2, which showed a high *V*_OC_ of 1.22 V with an acceptable PCE of 4.5% with P3HT as donor (Xiao B. et al., 2017a). Recently, a pyrene-fused perylene diimide acceptor synthesized by Li and Sun et al. can achieve a high *V*_OC_ of 1.21 V with a PCE of 5.10%, with the wide-bandgap polymer PBT1-EH as the donor (Zhan et al., 2017). Zhang et al. reported a combination of perylene monoimide (PMI)-based electron acceptor and a wide-bandgap polymer of PTZ1, which demonstrated a very high *V*_OC_ of 1.3 V with a PCE of 6% (Zhang Y. et al., 2017). However, in the above-cases, they used a trial-and-error procedure and there is no guideline how to choose suitable p-type polymer and n-type NFSMA combination to realize such a high *V*_OC_ and PCE simultaneously. Thus, finding a promising strategy to achieve a high *V*_OC_ without sacrificing the other impact factors is essential for practical application. Furthermore, this kind of OSCs with a high *V*_OC_ can also be used as a sub cell in the tandem devices to offer opportunities to realize a high *V*_OC_ beyond 2.0 V (Xu et al., 2018), which will supply high enough voltage for solar-energy-driven water splitting (Walter et al., 2010; Luo et al., 2014).

**Figure 2:**
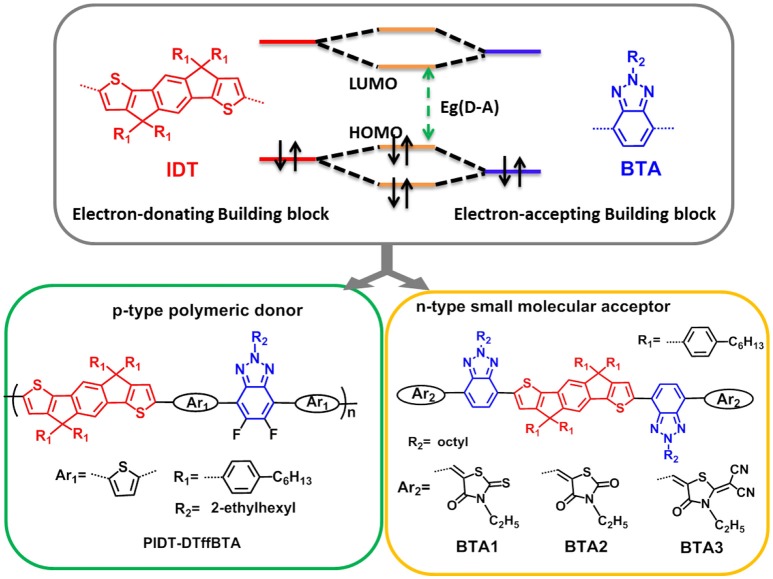
The basic design strategy of the photovoltaic materials containing the same electron-donating and accepting building blocks.

Based on the molecular orbital theory, for donor-acceptor (D-A)-type conjugated materials, the highest occupied molecular orbital (HOMO) and the lowest unoccupied molecular orbital (LUMO) energy level are mainly decided by the electron-donating and electron-withdrawing segments respectively. Thus, it may realize the close molecular energy levels by utilizing the same building blocks to build both donor and acceptor materials. By further slightly modulating the chemical structures, the enough LUMO-LUMO and HOMO-HOMO offsets could be realized to guarantee sufficient driving force for efficient hole and electron transfer and simultaneously result in an ultra-large Voc. In our previous work, we used “the Same-Acceptor-Strategy” (SAS), both p-type polymer of J61 and n-type small molecule of BTA3 contain the same electron-accepting unit of BTA, which could realize a high *V*oc of 1.15 V (Tang et al., 2017). In this paper, we further adopted this feasible strategy and chose the classic electron-donating unit of indacenodithiophene (IDT) and electron-accepting segment of benzotriazole (BTA) to construct the polymer donor and small molecular acceptors. The donor-acceptor (D-A) type copolymer of PIDT-DTffBTA, as shown in Figure 2, containing IDT as the donor unit and difluoro-substituted BTA as the acceptor unit and thiophene as π-bridge, was designed as the polymer donor. In addition, we utilized IDT and BTA to construct the conjugated backbone of the small molecular acceptors and fine-tuned the electron-withdrawing end-capped units to adjust their energy levels. In our previous work, we have proved that the energy levels of BTA-based small molecules could be fine-tuned and OSCs based P3HT: BTA1 (Xiao B. et al., 2017b), P3HT: BTA2 (Xiao B. et al., 2017a), and J61: BTA3 (Tang et al., 2017) could realize relatively higher *V*_OC_ of 1.02, 1.22, and 1.15 V, respectively.

Here, as expected, these devices based on PIDT-DTffBTA: BTA*x* (*x* = 1–3) as acceptors exhibited the reduced energy loss below 0.60 eV and the according *V*_OC_ in the range of 1.21–1.37 V were higher by nearly 0.3–0.5 V than that of [6,6]-phenyl-C_71_-butyric acid methyl ester (PC_71_BM) based device. Differently, the BTA2 and BTA1 based devices showed poor *J*_SC_ below 0.4 mA cm^−2^ while BTA3 gave a dramatically increased *J*_SC_ of 8.68 mA cm^−2^. It is remarkable that the achieved PCE of 5.67% in BTA3 based device is among the highest values for OSCs with a *V*_OC_ beyond 1.20 V reported so far. Our results provide a simple and feasible method to design both p-type and n-type photovoltaic materials for OSCs with high *V*_OC_ and PCE.

## Results and discussion

### Theoretical calculation

**Figure 3:**
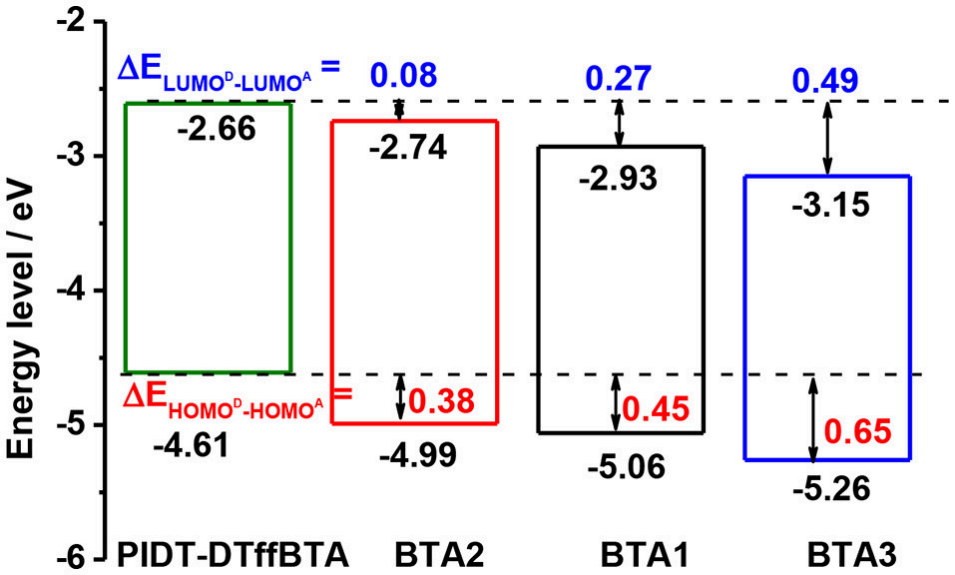
The simulated frontier molecular orbitals obtained by DFT calculation at B3LYP/6-31G(d) level.

Calculations with density functional theory (DFT) at the B3LYP/6-31G(d) level are firstly performed to compare the energy levels of these photovoltaic molecules. The polymers were replaced with the dimers of the repeating units and the long alkyls were replaced with methyl groups to simplify the calculations. As shown in Figure 3, the calculted LUMO/HOMO levels of PIDT-DTffBTA and BTA*x* (*x* = 1, 2, 3) are −2.66/−4.61, −2.93/−5.06, −2.74/−4.99, and −3.15/−5.26 eV, respectively. The LUMO offsets between PITD-DTffBTA and three acceptors (BTA2, BTA1 and BTA3) ($\Delta E_{LUMO^{D}-LUMO^{A}}$) are calculated to be 0.08, 0.27, and 0.49 eV, respectively, and the according HOMO offsets ($\Delta E_{HOMO^{D}-HOMO^{A}}$) are 0.38, 0.45, 0.65 eV, respectively. These results reveal that utilizing the same building blocks to construct both p-type polymeric donor and n-type small molecular acceptors has the potential to realize similar LUMO levels and give rise to high voltage. Modulating the end-capped units can help optimize the energy offsets to achieve the enough driving force for charge transfer and produce high photocurrent.

### Synthesis

The synthetic routes of the photovoltaic materials are depicted in Scheme S1. PIDT-DTffBTA was synthesized by Stille-coupling reaction between 4,7-bis-(5-bromothiophen-2-yl)-5,6-difluoro-2-octyl-2H-benzotriazole and (4,4,9,9-tetrakis(4-hexylphenyl)-4,9-dihydro-s-indaceno [1,2-b:5,6-b']dithiophene-2,7-diyl)bis(trimethylstannane). BTA*x* were synthesized by Stille-coupling reaction and a Knoevenagel condensation with a yield of 70–80%. The number-average molecular weight (M_n_) and polydispersity index (PDI) value of PIDT-DTffBTA are 62.8 kDa and 1.55, respectively, determined by gelpermeation chromatography (GPC) (see Figure S1). The temperature with the 5% weight loss (*T*_d_) of BTA*x* (*x* = 1–3) are 386, 405, and 396°C, respectively, measured with thermogravimetric analysis (TGA) (Figure S2**)**. All the materials are soluble in common organic solvents, such as chloroform (CF), chlorobenzene (CB), and *o*-dichlorobenzene (*o*-DCB).

### Optical properties

**Figure 4:**
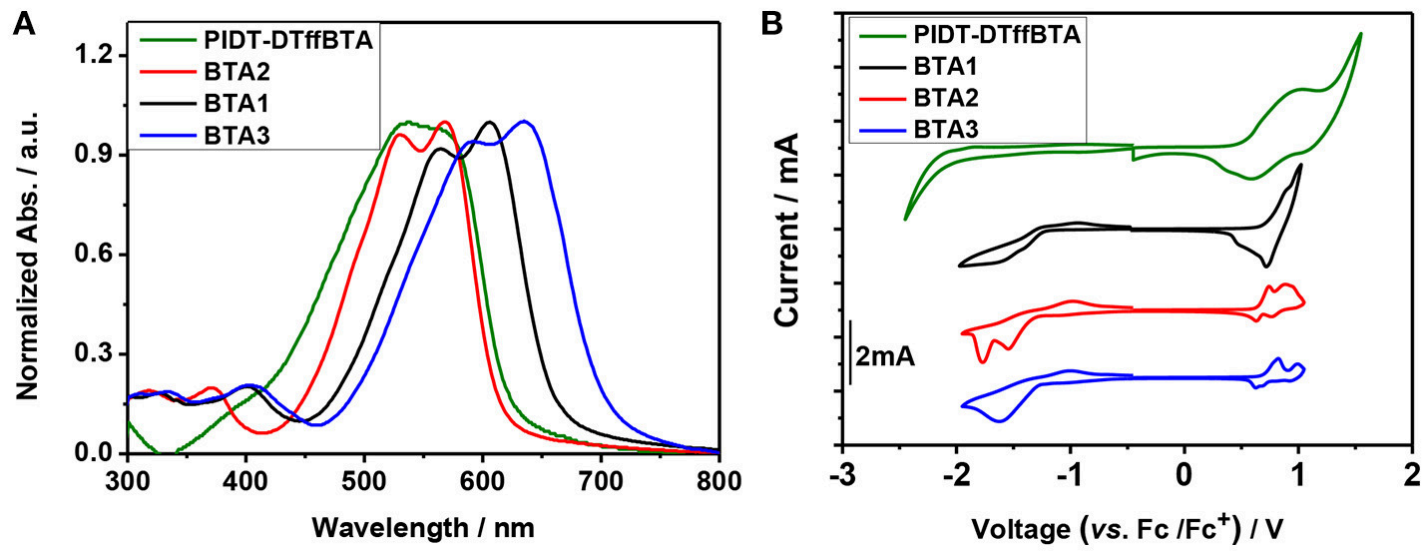
**(A)** The UV–vis absorption spectra of the pure films. **(B)** The CV curves of the four materials.

The UV–vis absorption spectra of the donor and acceptors in solution and films are shown in Figure S3 and Figure 4A, respectively, and their absorption characteristics are summarized in Table S1. The absorption of the BTA2 overlaps with that of PIDT-DTffBTA. The maximum absorption peaks red-shifts as the increase of the electron-withdrawing properties of the end-capped units. As a result, the absorption of BTA1 and BTA3 become more and more complementary to that of PITD-DTBTA, which would allow for an improved *J*_SC_ compared to BTA2 based devices. The optical band gaps ($E_{\text{g}}^{{}^{\circ}pt}$) of PIDT-DTffBTA and BTA*x* (*x* = 2, 1, 3) calculated from the film absorption onsets are ca. 1.96, 2.00, 1.87, 1.76 eV, respectively.

The molecular energy levels of these four materials are determined by electrochemical cyclic voltammetry (CV, Figure 4B). The according results are listed in Table S1. Calculated with their onset oxidation potentials, the HOMO levels of PIDT-DTffBTA and BTA*x* (*x* = 2, 1, 3) are −5.34, −5.43, −5.46, and −5.49 eV, respectively. The LUMO levels are estimated to be −3.38, −3.43, −3.59, and −3.73 eV, respectively, by adding the optical bandgap to their HOMO levels. $\Delta E_{HOMO^{D}-HOMO^{A}}$ between the donor and the acceptors (BTA2, 1, 3) are 0.09, 0.12, 0.15 eV, respectively, and $\Delta E_{LUMO^{D}-LUMO^{A}}$ are respectively 0.05, 0.21, 0.35 eV. As expected, the very small $\Delta E_{LUMO^{D}-LUMO^{A}}$ produce the ultar-high offsets between the HOMO level of the donor and the LUMO level of the acceptor, giving a chance to achieve the ultra-hight *V*_OC_ (Armstrong et al., 2009).

### Photovoltaic device performance

**Figure 5:**
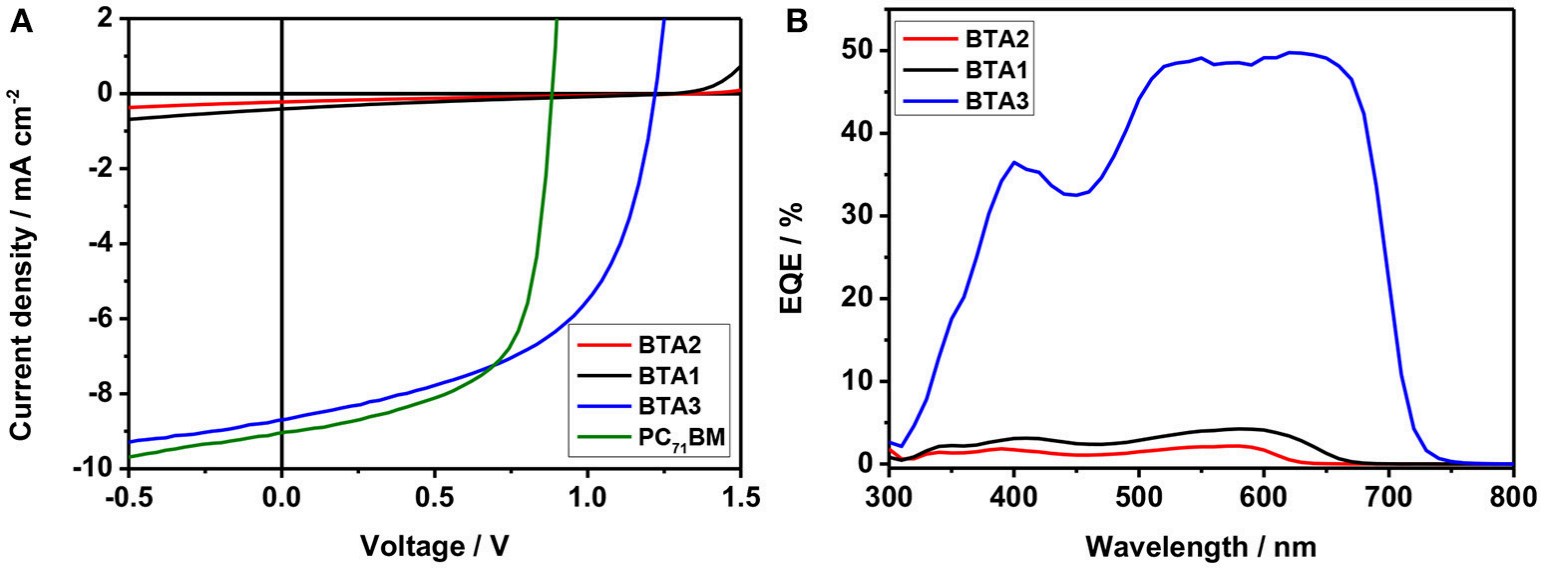
**(A)** *J*-*V* curves and **(B)** EQE curves of the optimal devices under the illumination of AM 1.5 G, 100 mW cm^−2^.

To investigate the photovoltaic properties, photovoltaic devices are fabricated with a conventional device configuration of indium tin oxide (ITO)/ poly(3,4-ethylenedioxythiophene): poly(styrenesulfonate)(PEDOT:PSS)/PIDT-DTffBTA: BTA*x* (*x* = 1–3)/Ca/Al. The optimized photovoltaic characteristics are listed in Table 1 and the optimal current density–voltage (*J*–*V*) curves and the corresponding external quantum efficiency (EQE) spectra are displayed in Figure 5. The detail optimization conditions are shown in Tables S2–S7. The device using the PIDT-DTffBTA: BTA1 and PIDT-DTffBTA: BTA2 blend (1:1 in wt %) with thermal annealing show nearly no performance with a PCE of 0.12 and 0.07%, respectively, which is likely due to their very high-lying LUMO, resulting in insufficient charge transfer from polymer to acceptor. Under the same conditions, the BTA3-based device (1:3 in wt %) exhibits improved solar cell performance with a PCE of 2.61%, which may be attributed to the decreased LUMO level. After solvent annealing, the devices using PIDT-DTffBTA: BTA3 show the highest PCE of 5.67% with the increased FF and *J*_SC_. It has been seen that after solvent annealing, the strong aggregation (Figure S4) can enhance domain purity and further improve charge transport in the active layer (see farther below), giving rise to the improved *J*_SC_ and FF.

**Table 1:** The photovoltaic characteristics of the PIDT-DTffBTA based OSCs.

**Acceptors**	***V*_OC_ [V]**	***J_*SC*_* [mA cm^−2^]**	**FF**	**PCE [%]**	**μ_h_ [cm^2^ V^−1^ s^−1^]**	**μ_e_ [cm^2^ V^−1^ s^−1^]**
BTA2	1.37 1.34 ± 0.02	0.21 0.21 ± 0.03	0.23 0.23 ± 0.02	0.07 0.065 ± 0.01	3.50 × 10^−5^	2.43 × 10^−7^
BTA1	1.27 1.27 ± 0.02	0.43 0.42 ± 0.00	0.22 0.22 ± 0.001	0.12 0.12 ± 0.00	4.96 × 10^−5^	4.21 × 10^−7^
BTA3	1.21 1.21 ± 0.00	8.68 8.69 ± 0.09	0.54 0.53 ± 0.01	5.67 5.63 ± 0.02	1.21 × 10^−4^	1.12 × 10^−5^
PC_71_BM	0.88 0.88 ± 0.01	9.04 8.88 ± 0.12	0.63 0.63 ± 0.01	5.01 4.93 ± 0.06	–	–
ITIC	0.94 0.945 ± 0.01	9.96 9.67 ± 0.06	54.04 53.65 ± 0.24	5.06 4.90 ± 0.03	–	–

As expected, all of the three devices show high *V*_OC_ (> 1.2 V), which are much higher than that of PIDT-DTffBTA: PC_71_BM ([6,6]-phenyl C_71_ butyric acid methyl ester) based device (*V*_OC_ = 0.88 V) and PIDT-DTffBTA: ITIC based device (*V*_OC_ = 0.94 V). The *V*_OC_ of the devices increase from 1.21, 1.27 to 1.37 V in the order of BTA3, BTA1, and BTA2. The energy loss values for the OSCs of PIDT-DTffBTA: BTA*x* (*x* = 2, 1, 3) are calculated to be 0.59, 0.60, and 0.55 eV, respectively, which is a result of the high *V*_OC_. Interestingly, the *J*_SC_ for PIDT-DTffBTA: BTA2 and PIDT-DTffBTA: BTA1 based devices are very low (<0.5 mA cm^2^), while the one for PIDT-DTffBTA:BTA3 based device dramatically reaches up to 8.68 mA cm^−2^, close to the value in PIDT-DTffBTA: PC_71_BM based device (9.06 mA cm^−2^). Accordingly, the maximum EQE values of PIDT-DTffBTA: BTA2 and PIDT-DTffBTA: BTA1 based devices are below 5%, while the one of PIDT-DTffBTA: BTA3 based device reaches up to 50%. The according current density obtained by the integration of the EQE curves are 0.24, 0.54, 8.85 mA cm^−2^, respectively, which are consistent with the *J*_SC_ values from the *J–V* curves within 5% error. Therefore, the OSCs based on PIDT-DTffBTA: BTA3 exhibits the best performance with a maximal PCE of 5.67%, which is among the highest values reported in the literature to date for NF OSCs with *V*_OC_ >1.20V.

**Figure 6:**
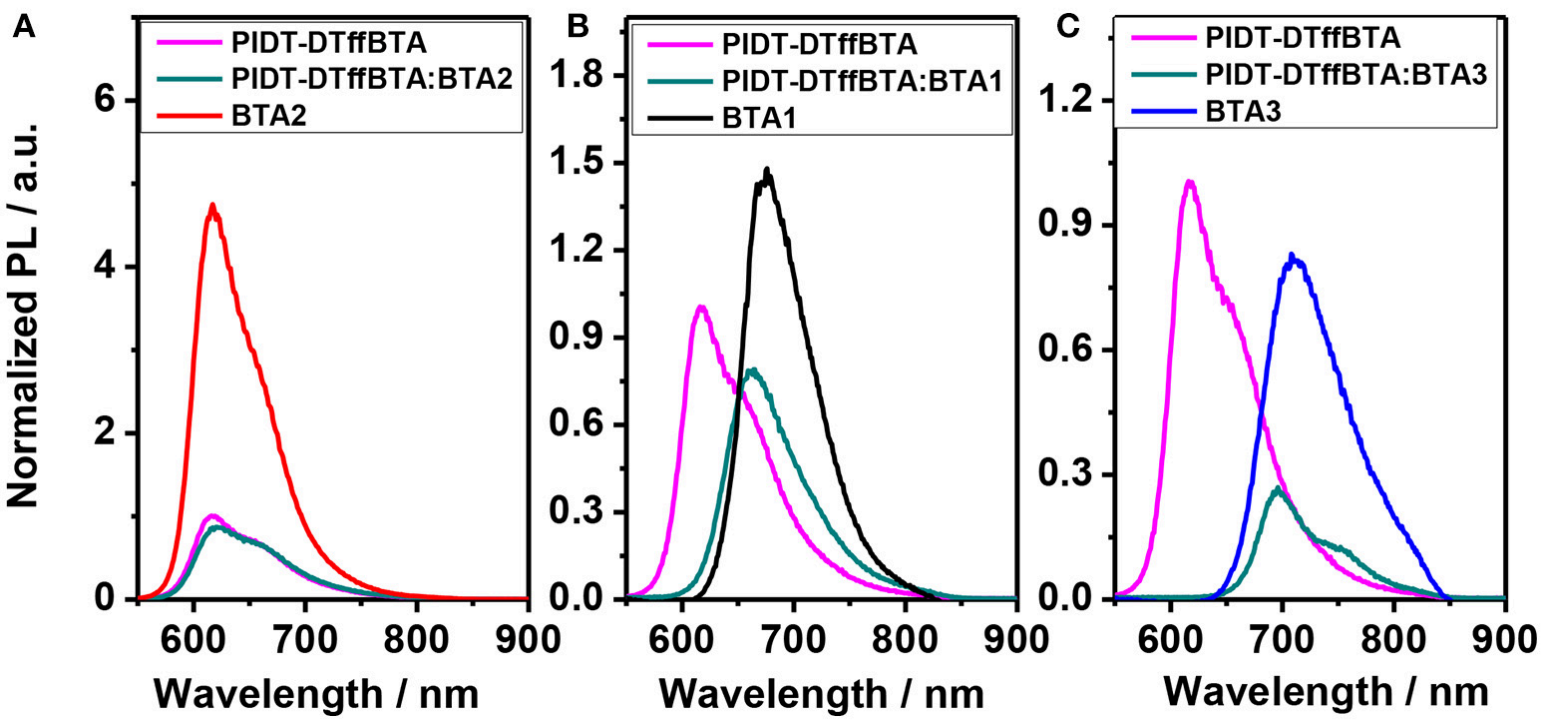
The photoluminescence spectra of neat PIDT-DTffBTA, acceptor films and BHJ blend films with the D/A ratio of 1:1: **(A)** BTA2; **(B)** BTA1; **(C)** BTA3. Note: all the films are excited at 480 nm.

**Figure 7:**
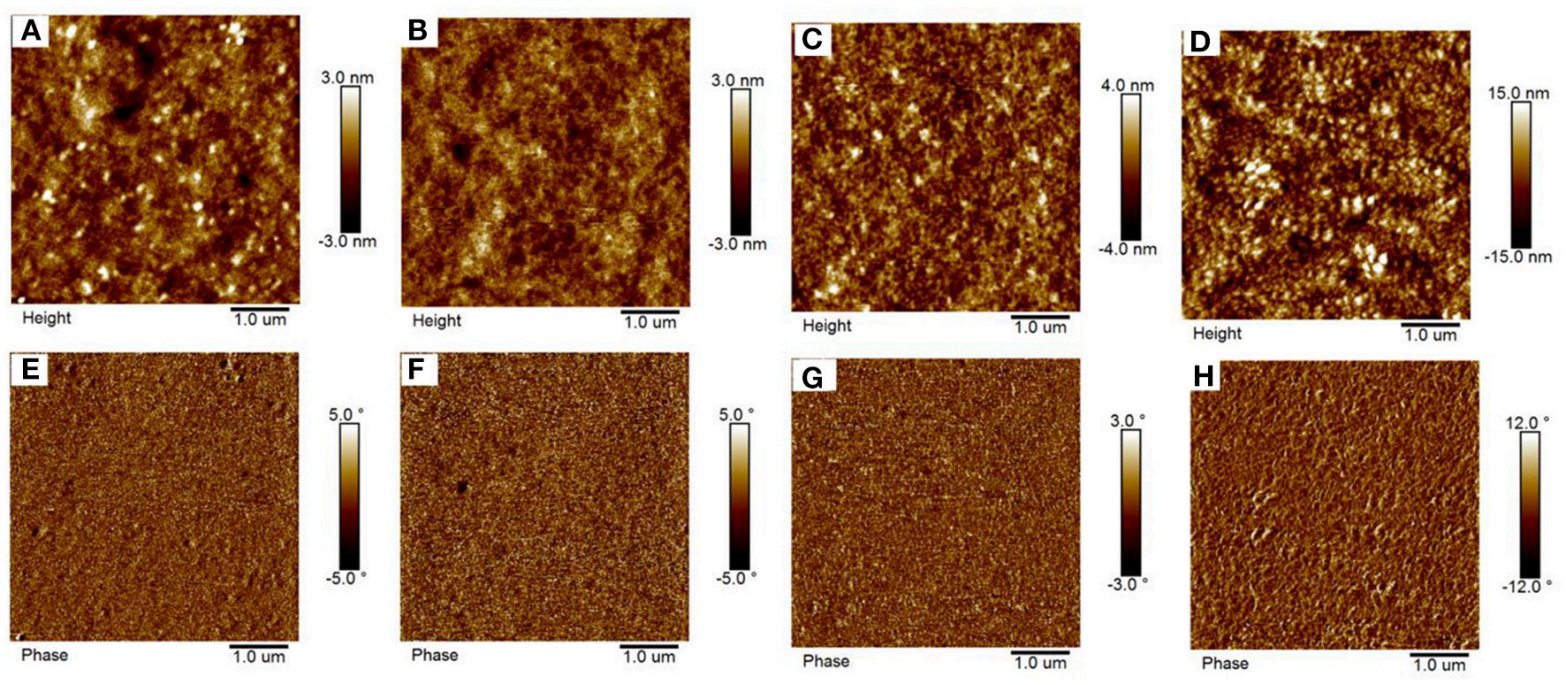
**(A–C)** The height images for the PIDT-DTffBTA:BTA2, PIDT-DTffBTA:BTA1, and PIDT-DTffBTA:BTA3 blend films without solvent annealing, respectively; **(E–G)** The phase images for the PIDT-DTffBTA:BTA2, PIDT-DTffBTA:BTA1, and PIDT-DTffBTA:BTA3 blend films without solvent annealing, respectively; **(D,H)** Is the height and phase images for the PIDT-DTffBTA:BTA3 blend films with solvent annealing, respectively.

To study the cause of the different *J*_SC_, we first investigate the exciton generation and separation by measuring the photoluminescence (PL) in these blends. As shown in Figure 6A, at the excitation wavelength of 480 nm, emission from the blend with BTA2 is close to the emission of the pristine PIDT-DTffBTA film and the PL quenching efficiency is only 16% (Li Z. et al., 2016). The inefficient quenching of PL indicates the excitons quick recombination rather than efficient separation. The shape of PL from the blend with BTA1 is close to the emission of the pristine BTA1 film but the polymer PL quenching efficiency significantly raises up to 88%, indicating that excitons initially generated on the PIDT-DTffBTA can transfer to BTA1 in high yield but they are inefficiently quenched by the heterojunction (Hoke et al., 2013). Considering the fine film morphology (as shown in Figure 7), the inefficient quenching of PL in PIDT-DTffBTA:BTA2 and PIDT-DTffBTA:BTA1 is owing to the too small $\Delta E_{LUMO^{D}-LUMO^{A}}$ (0.05 eV) and $\Delta E_{HOMO^{D}-HOMO^{A}}$ (0.12 eV), respectively. The too small energy offsets could reduce the overall exciton dissociation efficiency and create exergonic pathways for charge recombination of holes in PIDT-DTffBTA or electrons in BTA1, obviously increasing the voltage loss. As the increase of $\Delta E_{LUMO^{D}-LUMO^{A}}$ and $\Delta E_{HOMO^{D}-HOMO^{A}}$, the driving force for the hole and electron tranfer are obviously improved. Hence, BTA1 shows similar results with BTA2 but BTA3 can completely quench the PL of PIDT-DTffBTA with a quenching efficiency of ~100% and the luminous efficiency of BTA3 in the blend is as low as 25%, as shown in Figures 6B,C. The high PL quenching efficiency of both PIDT-DTffBTA and BTA3 suggests the improved electron and hole transfer in BTA3-based devices, which can partially explain its high photocurrent. Though *J*_SC_ and FF can be improved, they still below 9 mA/cm^2^ and 0.60, respectively, which may be attributed to its incomplete fluorescence quenching of BTA3, resulting in the modest hole transfer from BTA3 to polymer. Besides, time resolved photoluminescence measurements were also performed on neat and blended films with the same excited wavelength (Figure S5). All the time-resolved PL (TRPL) lifetime data fitted with bi-exponential decay model are summarized in Table 2. The fast decay is related to the exciton dissociation due to charge transfer at the donor/acceptor interfaces. The little changes in τ and *f* for the PIDT-DTffBTA:BTA2 and PIDT-DTffBTA:BTA1 blend films can be attributed to the poor exciton separation arising from the limitation of the small driving force. Moreover, the exciton lifetimes of PIDT-DTffBTA:BTA3 based blend film is clearly quenched as compared to neat films, suggesting both efficient hole and electron transfer at the donor/acceptor interface. Compared to the other two control device, the well improved exciton dissociation efficiency may explain part of the remarkably increased *J*_SC_ in the PIDT-DTffBTA:BTA3 based device. The PL quenching behavior of the TRPL measurements agrees well with the steady-state PL measurements discussed above.

**Table 2:** Fitting parameters of PL decay dynamics of the various films with excitation at 450 nm.

**Films**	**τ_1_ [ps]**	***f*_1_ [%]**	**τ_2_ [ns]**	***f*_2_ [%]**
PIDT-DTffBTA	402	98.84	6.01	1.16
BTA1	565	98.66	5.69	1.34
BTA2	432	96.13	2.53	3.87
BTA3	807	94.94	4.26	5.06
PIDT-DTffBTA:BTA1	432	95.60	3.28	4.40
PIDT-DTffBTA:BTA2	426	97.83	4.92	2.17
PIDT-DTffBTA:BTA3	317	93.44	3.16	6.56

Atom force microscope (AFM) is performed to investigate if the surface morphologies of the blend films play a role in the different *J*_SC_ and FF. As shown in the height images (Figures 7A–C), the PIDT-DTffBTA:BTA2, PIDT-DTffBTA: BTA1, and PIDT-DTffBTA: BTA3 based films without solvent annealing exhibit smooth and uniform surface morphologies with small root-mean-square roughness (RMS) of 0.95, 0.79, and 0.87 nm, respectively. Interestingly, the RMS in PIDT-DTffBTA: BTA3 based film increases up to 5.29 nm after solvent annealing (Figure 7D), which may be a result of the enhanced intermolecular aggregation effect (Figure S4) (Zhong et al., 2017) and increased crystallinity of BTA3 in the blend films (Figure S6) (Li et al., 2013). As shown in the phase images (Figures 7E–G), it is clear that all of the blend films exhibit interpenetrating networks with different domain sizes. The three blends of PIDT-DTffBTA: BTA*x* (*x* = 1–3) without solvent annealing show thinner domain sizes below 10 nm. While the blend of PIDT-DTffBTA and BTA3 shows the sufficient phase separation behavior with the domain size of 40–50 nm after solvent annealing (Figure 7H), which is beneficial to the efficient charge separation and transport, giving rise to the reduced recombination loss and improved *J*_SC_ and FF.

At last, the space charge limited current (SCLC) method is applied to measure the carriers mobilities (Figure S7). As listed in Table 1, the carriers mobilities observed in both PIDT-DTffBTA: BTA2 and PIDT-DTffBTA: BTA1 based devices are very low, on the order of only 10^−5^ cm^2^ V^−1^ s^−1^ for hole mobility (μ_h_) and 10^−7^ cm^2^ V^−1^ s^−1^ for electron mobility (μ_e_). These low and imbalance mobilities could result in the low FF observed in these devices (Earmme et al., 2013; Meng et al., 2015). The μ_h_ of BTA3 based device is 3–6 times higher than that of two other devices while the μ_e_ is higher about 2 order of magnitude, which is attributed to the dominant face-on orientation (Figure S6). As a result, the transport of holes and electrons in the PIDT-DTffBTA: BTA3 based device is faster and more balanced, which can effectively prevent the accumulation of charge and achieve higher FF and *J*_SC_.

## Conclusions

In this work, we applied a new design concept to construct the OSCs with ultrahigh *V*_OC_, utilizing the same building blocks (indacenodithiophene and benzotriazole) to design both p-type polymeric donor and n-type small molecular acceptors. The resulted non-fullerene acceptors showed high-lying LUMO levels, close to that of the donor polymer. With small voltage loss (0.55–0.60 V), all of the three devices show ultra-high *V*_OC_ (1.21–1.37 V). With the increase of the ability of attracting electron of acceptor, PIDT-DTffBTA: BTA3 device possesses the large driving force for efficient electron and hole transfer and yields a dramatically increased *J*_SC_. The achieved PCE of 5.67% is among the highest values for NF OSCs with a *V*_OC_ >1.20 V reported so far (as shown in Table S8). The results here offer a new method to construct the promising OSCs with ultra-high *V*_OC_, which could contribute to further improve the performance of the OSCs.

## Author contributions

Device fabrication and photovoltaic performance studies were carried out by AT and JY. Materials synthesis was carried out by BX and JL. EZ, FC, and XW contributed to project planning and discussions. EZ had the idea, led the project, and prepared the manuscript. All authors contributed to the manuscript preparation.

### Conflict of interest statement

The authors declare that the research was conducted in the absence of any commercial or financial relationships that could be construed as a potential conflict of interest.
