# Supplementary material for: Utilizing Benzotriazole and Indacenodithiophene Units to Construct Both Polymeric Donor and Small Molecular Acceptors to Realize Organic Solar Cells With High Open-Circuit Voltages Beyond 1.2 V
Source: Front Chem. 2018 May 1;6:147. doi: 10.3389/fchem.2018.00147 (PMC5938601; doi:10.3389/fchem.2018.00147)
Supplement: Supplementary file 1 [file Data_Sheet_1.docx]

**Supporting Information**

**Utilizing Benzotriazole and Indacenodithiophene Units to Construct both Polymeric Donor and Small Molecular Acceptors to Realize Organic Solar Cells with High Open-Circuit Voltages beyond 1.2 V**

Ailing Tang ^1^, Fan Chen^1,2^, Bo Xiao^1,2^, Jing Yang^1,2^, Jianfeng Li^1, 2^ , Xiaochen Wang^1^, Erjun Zhou^*1^

^1^CAS Key Laboratory of Nanosystem and Hierarchical Fabrication, CAS Center for Excellence in Nanoscience, National Center for Nanoscience and Technology, Beijing 100190, P. R. China.

^2^ University of Chinese Academy of Sciences, Beijing 100049, P. R. China.

E-mail: [zhouej@nanoctr.cn](mailto:zhouej@nanoctr.cn)

**Experiment Section**

**Instrumentation**

Molecular weights of polymers were determined by gel-permeation chromatography (GPC) using a narrow standard and o-dichlorobenzene (o-DCB) as the eluent at 140 °C. ^1^H NMR spectra was obtained using a Bruker Advance III 400 (400 MHz) nuclear magnetic resonance (NMR) spectroscope. Mass spectra (MALDI-TOF) were carried on a Micromass GCT-MS spectrometer. Lambda 950 spectrophotometer was used to characterize the UV–vis spectra. PL spectra were measured with Nanolog FL3-iHR320. Electrochemical CV was conducted with a Pt disk coated with a molecular film, a Pt wire, and an Ag/AgCl electrode acting as the working, counter, and reference electrodes, respectively, in a 0.1 mol L^−1^ tetrabutylammonium phosphorus hexafluoride (Bu_4_NPF_6_) acetonitrile solution, on an electrochemical workstation. The *J*−*V* curves were identified in air with a Keithley 2420 source measure unit. The photocurrent was measured under illumination using an Oriel Newport 150W solar simulator (AM 1.5G), and the light intensity was calibrated with a Newport reference detector (Oriel PN 91150 V). The EQE measurements of the devices were performed in air with an Oriel Newport system (Model 66902). Kla-TencorAlpha-StepD-120 Stylus Profiler was utilized to measure the thickness of the active layer. AFM images were obtained on a Multimode Digital Instrument in tapping mode. The samples were prepared with the optimized device fabrication conditions. Two-dimensional grazing incidence X-ray diffraction (2D-GIXD) analyses were measured at the SPring-8 on beamline BL46XU. The sample was irradiated at a fixed angle on the order of 0.12° through a Huber diffractometer with an X-ray energy of 12.39 keV (*λ* = 1 Å), and diffracted X-rays were captured at an exposure time of 1 s by a two-dimensional detector (PILATUS 300K) located at a distance of *L*=174 mm from the sample.

**Photovoltaic device fabrication**

ITO/PEDOT:PSS/PIDT-DTffBTA:BTA*x* /Ca/Al. A thin layer of PEDOT:PSS (30 nm, Baytron PH1000) was spin-cast on pre-cleaned ITO-coated glass at 3000 rpm. After baking at 150 °C for 20 min, the substrates were transferred into glovebox. Optimized devices were prepared under the following conditions. The donor and acceptor was dissolved in ODCB for 3 hour at 80 °C and then the active layers was spin-coated from the above solution. The thickness of the photoactive layer is in the range of 90−100 nm. The effects of solvents, additive concentrations, solvent annealing, thermal annealing temperature, and blend ratios on device performance were also examined. Finally, a Ca (20 nm)/Al (80nm) metal top electrode was thermal evaporated onto the active layer under about 2×10^-6^ mbar. The active area of the device was 0.04 cm^2^ defined by shadow mask.

**Carrier Mobilities.**

The space charge limited current (SCLC) method was applied to investigate the hole and electron mobility of the devices. The hole only mobility of the blend films was measured with the device structure of ITO/PEDOT:PSS/active layer/Au (80 nm) and the electron mobility of the blends was measured with the device structure of ITO/TiO_x_/active layer/Al (80 nm).

The SCLC model is described by modified Mott-Gurney law:

$$J=(9/8)\varepsilon_{0}\varepsilon_{r}\mu(V^{2}/L^{3})exp[0.89\beta\left( V/L \right)^{0.5}]$$

where *J* stands for current density, *ε*_0_ is the permittivity of free space (8.85×10^−12^CV^−1^ m^−1^), *ε*_r_ is the relative dielectric constant of the transport medium (assuming that 3.0), *μ* is the carrier mobility, *V* is the internal potential in the device and *L* is the thickness of the active layer, β is the field activation factor.

The thickness of the active layers are 87 nm, 79 nm and 105 nm for PIDT-DTffBTA:BTA1, PIDT-DTffBTA:BTA2 and PIDT-DTffBTA:BTA3, respectively.

**Materials and Synthesis.**

All the reagents were purchased from Alfa, Aldrich, TCI or Wako and used without further purification. Solvents were bought from the Beijing Chemical Plant. The precursor 1 and 4,7-bis-(5-bromothiophen-2-yl)-5,6-difluoro-2-octyl-2H-benzotriazole were bought from the regent plant (their NMR and MALDI-TOF was shown in Figure S7-S10 and S12-13). BTA1-3 were synthesized according the routes that we have previously reported. [1-3]

Scheme S1. The synthetic route of PIDT-DTffBTA.

**The synthesis of PIDT-DTffBTA**

(4,4,9,9-tetrakis(4-hexylphenyl)-4,9-dihydro-s-indaceno[1,2-b:5,6-b’]dithiophene-2,7diyl)bis(trimethylstannane) (0.5 mmol, 616 mg) was added into a two necked flack and under the protection of nitrogen, 4,7-bis-(5-bromothiophen-2-yl)-5,6-difluoro-2-octyl-2H-benzotriazole (0.5 mmol, 294 mg) and degassed toluene (20 mL) was added in. The mixture was purged with nitrogen for 10 min and then Pd(PPh_3_)_4_ (30 mg) was put in. The mixture was flushed with nitrogen for another 10 min and then refluxed for 48 hours. After cooling to room temperature, the mixture was poured into methanol (MeOH, 300 mL) and stirred for 10 minutes, then filtered. Then the solid was subjected to Soxhlet extraction with MeOH, hexane, DCM and CF. The polymer was recovered as a solid from the CF fraction by evaporation. The yield of this part was about 19%. The yield of the polymer from the DCM fraction was 72%. ^1^H NMR spectrum of polymer in CDCl_3_ was shown in Figure S11. GPC: M_n_ = 62.8 kg mol^-1^, PDI = 1.55.


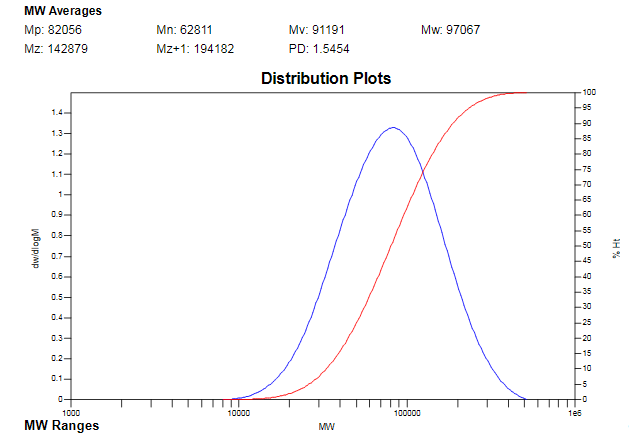


Figure S1. The GPC chromatograms of PIDT-DTffBTA measured in ODCB.

Figure S2. TGA curves of BTA1-3 with a scanning rate of 10 °C min^-1^ under an atmosphere of N_2_.

Figure S3. The UV−vis absorption spectra of the photovoltaic materials in solution.

Figure S4. The UV−vis absorption spectra of the blend films.

Figure S5. The normalized time-resolved PL spectra for the blend films with the excitation wavelength at 450 nm a) PIDT-DTffBTA:BTA1 and b) PIDT-DTffBTA:BTA2 and c) PIDT-DTffBTA:BTA3. For comparison, kinetics of the neat films of PIDT-DTffBTA, BTA1, BTA2, and BTA3 are also measured under the same condition.


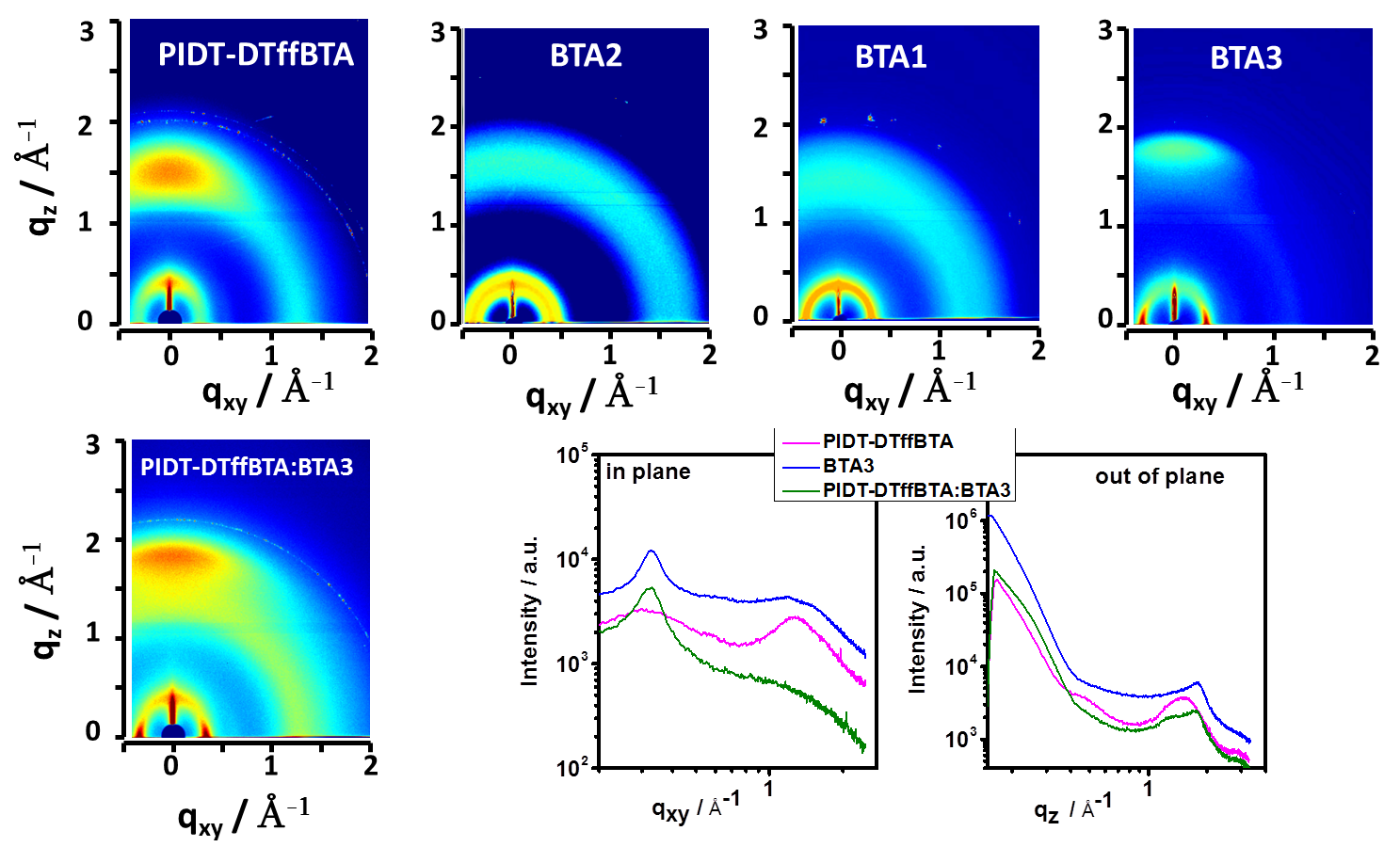


Figure S6. 2D GIWAXS patterns of the neat films and the PIDT-DTffBTA:BTA3 based blend film and the line profiles of the scattering images for PIDT-BTffBTA, BTA3 and PIDT-DTffBTA:BTA3 films in the out-of-plane (*q*_z_) and the in-plane (*q*_xy_) directions.

Figure S7. (a) Hole and (b) electron mobility plots from SCLC methods. Note: the red rhombus represents BTA2; the black del operator represents BTA1; the blue sphere is the BTA3.

**Table S1**. The optical properties and energy levels of these photovoltaic materials.

| Compounds | solutions | | blend films | | | E_HOMO_ *^a^*  (eV) | E_LUMO_ *^a^*  (eV) | E_LUMO_ *^b^*  (eV) |
| --- | --- | --- | --- | --- | --- | --- | --- | --- |
|  | ε_max_ (×10^5^L/mol/cm) | λ_max_  (nm) | λ_max_  (nm) | λ_onset_  (nm) | E_g_  (eV) |  |  |  |
| PIDT-DTffBTA | 0.79 | 561 | 534,  566 | 628 | 1.96 | -5.34 | -3.43 | -3.38 |
| BTA2 | 1.10 | 557 | 530,  561 | 620 | 2.00 | -5.43 | -3.48 | -3.43 |
| BTA1 | 1.13 | 592 | 562,  605 | 663 | 1.87 | -5.46 | -3.55 | -3.59 |
| BTA3 | 1.14 | 613 | 593,  636 | 704 | 1.76 | -5.49 | -3.57 | -3.73 |

*^a^* calculated based on the equations E_HOMO_ = − e ( *E*_ox_+ 4.80) (eV); E_LUMO_ = − e ( E_red_ + 4.80) (eV). *^b^* calculated by adding optical bandgap to their HOMO levels.

**Photovoltaic performance**

**Table S2**. The photovoltaic performance of PIDT-DTffBTA:BTA*_x_*(*x*=1-3) under different solvent with 1:1 (D:A)

| devices | solvent | *V*_OC_ (V) *^a^* | *J*_SC_(mA cm^-2^) | FF (%) | PCE (%) |
| --- | --- | --- | --- | --- | --- |
| PIDT-DTFFBTA:BTA3 | CF | 1.23 | 2.46 | 26.74 | 0.81 |
|  | CB | 1.24 | 3.24 | 28.12 | 1.13 |
|  | *o*-DCB | 1.23 | 3.46 | 29.40 | 1.25 |
| PIDT-DTFFBTA:BTA2 | CF | 1.34 | 0.12 | 26.01 | 0.043 |
|  | *o*-DCB | 1.37 | 0.23 | 22.39 | 0.071 |
| PIDT-DTFFBTA:BTA1 | *o*-DCB | 1.27 | 0.42 | 22.48 | 0.12 |

**Table S3**. The photovoltaic performance of PIDT-DTffBTA:BTA*_x_*(*x*=1-3) under different D/A ratios after thermal annealing 150 °C, 10 min.

| devices | D:A | *V*_OC_(V) | *J_SC_* (mA cm^-2^) | FF (%) | PCE (%) |
| --- | --- | --- | --- | --- | --- |
| PIDT-DTffBTA:BTA3 | 1:1.5 | 1.23 | 5.08 | 33.58 | 2.10 |
|  | 1:2 | 1.23 | 5.50 | 35.04 | 2.37 |
|  | 1:3 | 1.23 | 5.90 | 35.98 | 2.61 |
|  | 1:4 | 1.22 | 5.56 | 36.56 | 2.48 |
| PIDT-DTffBTA:BTA1 | 1:1 | 1.27 | 0.42 | 22.48 | 0.12 |
|  | 1:3 | 1.20 | 0.41 | 24.67 | 0.12 |
| PIDT-DTffBTA:BTA2 | 1:1 | 1.37 | 0.23 | 22.39 | 0.071 |
|  | 1:3 | 1.35 | 0.11 | 23.38 | 0.040 |

**Table S4**. The photovoltaic performance of PIDT-DTffBTA:BTA*_x_*(*x*=1-3) under different thermal annealing temperature.

| devices | annealing temperature (°C) | *V*_OC_ (V) *^a^* | *J*_SC_(mA cm^-2^) | FF (%) | PCE (%) |
| --- | --- | --- | --- | --- | --- |
| PIDT-DTffBTA:BTA3 | r.t. | 1.23 | 6.77 | 50.21 | 4.18 |
|  | 120 | 1.24 | 6.33 | 50.43 | 3.96 |
|  | 150 | 1.22 | 7.07 | 53.12 | 4.58 |
|  | 170 | 1.22 | 7.08 | 52.92 | 4.57 |

**Table S5**. The photovoltaic performance of PIDT-DTffBTA:BTA*_x_*(*x*=1-3) under different D/A ratios after solvent and thermal annealing at 150 °C, 10 min.

| devices | D/A | *V*_OC_ (V) | *J*_SC_(mA cm^-2^) | FF (%) | PCE (%) |
| --- | --- | --- | --- | --- | --- |
| PIDT-DTffBTA:BTA3 | 1:1 | 1.22 | 5.45 | 44.70 | 2.97 |
|  | 1:1.5 | 1.22 | 7.13 | 50.84 | 4.42 |
|  | 1:3 | 1.21 | 8.63 | 53.84 | 5.62 |
| PIDT-DTffBTA:BTA1 | 1:1 | 1.27 | 0.42 | 22.48 | 0.12 |
|  | 1:3 | 1.20 | 0.41 | 24.67 | 0.12 |
| PIDT-DTffBTA:BTA2 | 1:1 | 1.37 | 0.23 | 22.39 | 0.071 |
|  | 1:3 | 1.35 | 0.11 | 23.38 | 0.040 |
| PIDT-DTffBTA:ITIC  150 °C, 10 min and solvent annealing | 1.5:1 | 0.96 | 3.47 | 42.94 | 1.63 |
|  | 1:1 | 1500 | 0.97 | 6.02 | 49.63 |
|  | 1:1.5 | 0.96 | 8.27 | 55.30 | 5.01 |
|  | 1:3 | 0.94 | 8.09 | 57.46 | 4.99 |

**Table S6**. The photovoltaic performance of PIDT-DTffBTA:BTA*_x_*(*x*=1-3) under different solvent additive at solvent annealing and 150 °C thermal annealing.

| devices | Solvent  additive | *V*_OC_ (V) *^a^* | *J*_SC_(mA cm^-2^) | FF (%) | PCE (%) |
| --- | --- | --- | --- | --- | --- |
| PIDT-DTffBTA:BTA3 | No | 1.21 | 8.71 | 53.80 | 5.67 |
|  | 1%DIO | 1.18 | 5.40 | 50.26 | 3.20 |
|  | 1%CN | 1.20 | 8.07 | 52.45 | 5.08 |
|  | 1%DPE | 1.21 | 8.48 | 52.63 | 5.40 |

**Table S7**. The photovoltaic performance of PIDT-DTffBTA:BTA*_x_* (*x*=1-3) under different film thickness.

| Devices | D:A | rpm | *V*_OC_ (V) | *J*_SC_(mA cm^-2^) | FF (%) | PCE (%) |
| --- | --- | --- | --- | --- | --- | --- |
| PIDT-DTffBTA:BTA3  ODCB  150 °C, 10min | 1:1.5  25mg/mL | 1000 | 1.22 | 4.26 | 32.98 | 2.06 |
|  |  | 1200 | 1.23 | 4.24 | 33.58 | 2.10 |
|  |  | 1500 | 1.23 | 4.18 | 33.12 | 2.05 |
|  | 1:2  30mg/mL | 1500 | 1.23 | 4.39 | 34.33 | 2.21 |
|  |  | 2000 | 1.23 | 4.58 | 35.04 | 2.37 |
|  |  | 2500 | 1.23 | 4.46 | 34.51 | 2.26 |
|  | 1:3  40mg/mL | 1500 | 1.22 | 4.06 | 35.60 | 2.11 |
|  |  | 2500 | 1.23 | 4.68 | 36.33 | 2.50 |
|  |  | 3000 | 1.23 | 4.67 | 35.35 | 2.44 |
|  |  | 3500 | 1.23 | 4.92 | 35.98 | 2.61 |
|  | 1:4  40mg/mL | 2500 | 1.22 | 4.55 | 36.49 | 2.44 |
|  |  | 3000 | 1.22 | 4.61 | 36.56 | 2.48 |
|  |  | 3500 | 1.22 | 4.51 | 36.05 | 2.40 |

**Table S8**. Eg, E_loss_, *V*_OC_, EQE_max_ and PCE for various polymer solar cells.

| polymer/Fullerence | Eg (eV) | E_loss_ (eV) | *V*_OC_ (V) | PCE (%) | EQE_max_ | References |
| --- | --- | --- | --- | --- | --- | --- |
| PCz:EV-BT | 2.40 | 1.01 | 1.39 | 0.69 | 0.26 | 4 |
| P3HT:PFDTBT-OC6 | 1.90 | 0.54 | 1.36 | 1.80 | 0.22 | 5 |
| P3HT:F4TBT4 | 1.90 | 0.64 | 1.26 | 4.12 | 0.45 | 6 |
| P3HT:BTA2 | 1.90 | 0.68 | 1.22 | 4.50 | 0.50 | 1 |
| P3HT:PF12TBT | 1.90 | 0.64 | 1.26 | 2.70 | - | 7 |
| P3HT:F8TBT | 1.90 | 0.55 | 1.35 | 1.87 | 0.20 | 8 |
| PCDTBT-C12:NI-T-NI | 1.97 | 0.67 | 1.30 | 2.01 | 0.30 | 9 |
| PCDTBT: P-BNBP-T | 1.80 | 0.50 | 1.30 | 3.20 | 0.39 | 10 |
| PBT1-EH: TPAPPDI | 1.88 | 0.67 | 1.21 | 5.10 | 0.41 | 11 |
| PBT1-EH: TPEPPDI | 1.96 | 0.72 | 1.24 | 2.24 | 0.32 | 11 |
| PTZ1:PMI-F-PMI | 1.97 | 0.67 | 1.30 | 6.00 | 0.58 | 12 |
| P3HT:SF-DPPEH |  | 0.69 | 1.1 | 3.63 | 0.41 | 13 |
| P3HT:F(DPP)2B2 | 1.82 | 0.64 | 1.18 | 3.17 | 0.26 | 14 |
| DR3TSBDT :DTBTF | 1.77 | 0.62 | 1.15 | 3.84 | 0.41 | 15 |
| P3HT : SF(DPPB)4 | 1.75 | 0.61 | 1.14 | 5.16 | 0.48 | 16 |
| P3HT:4D | 1.72 | 0.54 | 1.18 | 3.86 | 0.26 | 17 |
| PDCBT-F:IT-M | 1.59 | 0.46 | 1.13 | 6.6 | 0.5 | 18 |
| P3TEA:FTTB-PDI4 | 1.66 | 0.53 | 1.13 | 10.58 | 0.68 | 19 |
| PBDTS-TDZ:ITIC | 1.65 | 0.55 | 1.10 | 12.8 | 0.80 | 20 |
| PCz:EV-BT | 2.40 | 1.04 | 1.36 | 0.75 | 0.19 | 21 |
| PCDTBT-C12:SM4 | 1.97 | 0.73 | 1.24 | 0.13 | 0.03 | 22 |
| PCDTBT-C12:SM1 | 1.97 | 0.61 | 1.36 | 0.59 | 0.15 | 22 |
| PCDTBT-C12:SM2 | 1.97 | 0.78 | 1.19 | 0.34 | 0.08 | 22 |
| PCDTBT-C12:SM3 | 1.97 | 0.86 | 1.11 | 0.74 | 0.04 | 22 |
| P3ETA:SF-PDI_2_ | 1.72 | 0.61 | 1.11 | 9.50 | - | 23 |
| P(BTI-F):PCBM | 1.86 | 0.75 | 1.11 | 1.61 | - | 24 |
| PIDT-DTffBTA:BTA2 | 1.96 | 0.59 | 1.37 | 0.07 | 0.02 | our work |
| PIDT-DTffBTA:BTA1 | 1.87 | 0.60 | 1.27 | 0.12 | 0.05 | our work |
| PIDT-DTffBTA:BTA3 | 1.76 | 0.55 | 1.21 | 5.67 | 0.50 | our work |
| J61:BTA2 | 1.94 | 0.65 | 1.29 | 0.26 | 0.06 | 3 |
| J61:BTA1 | 1.87 | 0.63 | 1.24 | 3.02 | 0.34 | 3 |
| J61:BTA3 | 1.76 | 0.61 | 1.15 | 8.25 | 0.62 | 3 |

**Reference**

(1) Xiao, B.; Tang, A.; Yang, J.; Wei, Z.; Zhou, E. *ACS Macro Lett.* **2017**, 410-414.

(2) Xiao, B.; Tang, A.; Zhang, J.; Mahmood, A.; Wei, Z.; Zhou, E. *Adv. Energy Mater.* **2016**, 1602269.

(3) Tang, A., Xiao, B., Wang, Y., Gao, F., Tajima, K., Bin, H., Zhang, Z., Li, Y., Wei, Z., Zhou, E. *Adv. Func. Mater.* **2017,** DOI:10.1002/adfm.201704507.

(4). Ooi, Z. E.; Tam, T. L.; Shin, R. Y. C.; Chen, Z. K.; Kietzke, T.; Sellinger, A.; Baumgarten, M.; Mullen, K.; deMello, J. C. Solution Processable Bulk-Heterojunction Solar Cells Using a Small Molecule Acceptor. *J. Mater. Chem.* **2008,** *18*, 4619.

(5). Yang, Q. S., H.; Gao, B.; Wang, Y.; Fu, Y.; Yang, J.; Xie, Z.; Wang, L. ;. High Open-Circuit Voltage Polymer/Polymer Blend Solar Cells with a Polyfluorene Copolymer as the Electron Acceptor. *RSC Adv.* **2014,** *4*, 12579-12585.

(6). Fu, Y.; Wang, B.; Qu, J.; Wu, Y.; Ma, W.; Geng, Y.; Han, Y.; Xie, Z. Fullerene-Free Polymer Solar Cells with Open-Circuit Voltage above 1.2 V: Tuning Phase Separation Behavior with Oligomer to Replace Polymer Acceptor. *Adv. Funct. Mater.* **2016,** *26*, 5922-5929.

(7). Mori, D.; Benten, H.; Ohkita, H.; Ito, S.; Miyake, K. Polymer/Polymer Blend Solar Cells Improved by Using High-Molecular-Weight Fluorene-Based Copolymer as Electron Acceptor. *ACS Appl. Mater. Interfaces* **2012,** *4*, 3325-3329.

(8). Yu, W.; Yang, D.; Zhu, X.; Wang, X.; Tu, G.; Fan, D.; Zhang, J.; Li, C. Control of Nanomorphology in All-Polymer Solar Cells Via Assembling Nanoaggregation in a Mixed Solution. *ACS Appl. Mater. Interfaces* **2014,** *6*, 2350-2355.

(9). Zhang, X.; Zhang, J.; Lu, H.; Wu, J.; Li, G.; Li, C.; Li, S.; Bo, Z. A 1,8-Naphthalimide Based Small Molecular Acceptor for Polymer Solar Cells with High Open Circuit Voltage. *J. Mater. Chem. C* **2015,** *3*, 6979-6985.

(10). Ding, Z.; Long, X.; Meng, B.; Bai, K.; Dou, C.; Liu, J.; Wang, L. Polymer Solar Cells with Open-Circuit Voltage of 1.3 V Using Polymer Electron Acceptor with High Lumo Level. *Nano Energy* **2017,** *32*, 216-224.

(11). Zhan, X.; Xiong, W.; Gong, Y.; Liu, T.; Xie, Y.; Peng, Q.; Sun, Y.; Li, Z. Pyrene-Fused Perylene Diimides: New Building Blocks to Construct Non-Fullerene Acceptors with Extremely High Open-Circuit Voltages up to 1.26 V. *Solar RRL* **2017,** *1*, 1700123.

(12). Zhang, Y.; Guo, X.; Guo, B.; Su, W.; Zhang, M.; Li, Y. Nonfullerene Polymer Solar Cells Based on a Perylene Monoimide Acceptor with a High Open-Circuit Voltage of 1.3 V. *Adv. Funct. Mater.* **2017,** *27*, 1603892.

(13). Wu, X.-F.; Fu, W.-F.; Xu, Z.; Shi, M.; Liu, F.; Chen, H.-Z.; Wan, J.-H.; Russell, T. P. Spiro Linkage as an Alternative Strategy for Promising Nonfullerene Acceptors in Organic Solar Cells. *Adv. Funct. Mater.* **2015,** *25*, 5954-5966.

(14). Li, S.; Yan, J.; Li, C.-Z.; Liu, F.; Shi, M.; Chen, H.; Russell, T. P. A Non-Fullerene Electron Acceptor Modified by Thiophene-2-Carbonitrile for Solution-Processed Organic Solar Cells. *J. Mater. Chem. A* **2016,** *4*, 3777-3783.

(15). Ni, W.; Li, M.; Kan, B.; Liu, F.; Wan, X.; Zhang, Q.; Zhang, H.; Russell, T. P.; Chen, Y. Fullerene-Free Small Molecule Organic Solar Cells with a High Open Circuit Voltage of 1.15 V. *Chem. Commun. (Camb)* **2016,** *52*, 465-468.

(16). Li, S.; Liu, W.; Shi, M.; Mai, J.; Lau, T.-K.; Wan, J.; Lu, X.; Li, C.-Z.; Chen, H. A Spirobifluorene and Diketopyrrolopyrrole Moieties Based Non-Fullerene Acceptor for Efficient and Thermally Stable Polymer Solar Cells with High Open-Circuit Voltage. *Energy Environ. Sci.* **2016,** *9*, 604-610.

(17). A. Rananaware; A. Gupta; J. Li; A. Bilic; L. Jones; S. Bhargava; Bhosale, S. V. A Four-Directional Non-Fullerene Acceptor Based on Tetraphenylethylene and Diketopyrrolopyrrole Functionalities for Efficient Photovoltaic Devices with a High Open-Circuit Voltage of 1.18 V. *Chem. Commun.* **2016,** *52*, 8522-8525.

(18). Zhang, H.; Li, S.; Xu, B.; Yao, H.; Yang, B.; Hou, J. Fullerene-Free Polymer Solar Cell Based on a Polythiophene Derivative with an Unprecedented Energy Loss of Less Than 0.5 Ev. *J. Mater. Chem. A* **2016,** *4*, 18043-18049.

(19). Zhang, J.; Li, Y.; Huang, J.; Hu, H.; Zhang, G.; Ma, T.; Chow, P. C. Y.; Ade, H.; Pan, D.; Yan, H. Ring-Fusion of Perylene Diimide Acceptor Enabling Efficient Nonfullerene Organic Solar Cells with a Small Voltage Loss. *J. Am. Chem. Soc.* **2017,** *139*, 16092-16095.

(20). Xu, X.; Yu, T.; Bi, Z.; Ma, W.; Li, Y.; Peng, Q. Realizing over 13% Efficiency in Green-Solvent-Processed Nonfullerene Organic Solar Cells Enabled by 1,3,4-Thiadiazole-Based Wide-Bandgap Copolymers. *Adv. Mater.* **2018,** *30*, 1703973.

(21). Ooi, Z. E.; Tam, T. L.; Shin, R. Y. C.; Chen, Z. K.; Kietzke, T.; Sellinger, A.; Baumgarten, M.; Mullen, K.; deMello, J. C. Solution Processable Bulk-Heterojunction Solar Cells Using a Small Molecule Acceptor. *J. Mater. Chem.* **2008,** *18*, 4619.

(22). Zhang, J.; Zhang, X.; Xiao, H.; Li, G.; Liu, Y.; Li, C.; Huang, H.; Chen, X.; Bo, Z. 1,8-Naphthalimide-Based Planar Small Molecular Acceptor for Organic Solar Cells. *ACS Appl. Mater. Interfaces* **2016,** *8*, 5475-5483.

(23). Liu, J.; Chen, S.; Qian, D.; Gautam, B.; Yang, G.; Zhao, J.; Bergqvist, J.; Zhang, F.; Ma, W.; Ade, H.; Inganäs, O.; Gundogdu, K.; Gao, F.; Yan, H. Fast Charge Separation in a Non-Fullerene Organic Solar Cell with a Small Driving Force. *Nat. Energy* **2016,** *1*, 16089.

(24). Li, H.; Sun, S.; Mhaisalkar, S.; Zin, M. T.; Lam, Y. M.; Grimsdale, A. C. A High Voltage Solar Cell Using a Donor-Acceptor Conjugated Polymer Based on Pyrrolo[3,4-F]-2,1,3-Benzothiadiazole-5,7-Dione. *J. Mater. Chem. A* **2014,** *2*, 17925-17933.
